# Atypical Presentation of a Preexisting Pancreatic Pseudocyst with Glucagon-Like Peptide-1 Agonist as a Possible Trigger for Exacerbation: A Case Report

**DOI:** 10.7759/cureus.47559

**Published:** 2023-10-24

**Authors:** Murtajiz M Raza, Matthew R Logan, David L Stuart, Hrishi Patel

**Affiliations:** 1 Research, Avalon University School of Medicine, Willemstad, CUW; 2 Surgery, Beckley Appalachian Regional Healthcare (ARH) Hospital, Beckley, USA; 3 Internal Medicine, Beckley Appalachian Regional Healthcare (ARH) Hospital, Beckley, USA

**Keywords:** pancreatic pseudocyst, glucagon-like peptide-1 receptor agonist, glucagon-like peptide 1, pseudocyst of the pancreas, cystic mass of pancreas

## Abstract

Pancreatitis encompasses pancreatic tissue inflammation due to enzymatic autodigestion, leading to complications such as pancreatic pseudocysts. This report details a case in which the administration of glucagon-like peptide-1 (GLP-1) agonists rendered a regressing, asymptomatic pseudocyst symptomatic. We posit that, absent other triggers, GLP-1 agonists might exacerbate pseudocysts. This emphasizes timely diagnosis and proper management. Our investigation delves into patient-specific nuances, potential mechanistic insights, the need to study this phenomenon among a broader cohort, alternative pathologies, long-term consequences, and clinical ramifications in response to a shifting pseudocyst behavior.

## Introduction

Acute pancreatitis, resulting from inflammation and reversible pancreatic tissue damage, can lead to pancreatic pseudocysts. Its etiology encompasses factors like pancreatic ductal obstruction, alcohol abuse, genetic mutations, toxins, infections, diminished blood supply, and trauma [1]. Pseudocysts, collections of liquefied necrotic tissue enclosed by granulation tissue, lack an epithelial lining but are enzyme-rich. While many regress, larger ones can compress adjacent structures, causing symptoms and even perforation [2,3]. It is rare to see a shrinking pseudocyst cause symptoms in a patient. Glucagon-like peptide-1 (GLP-1) agonists have been associated with pancreatitis [4], covered in literature exploring their safety and rare complications; however, can they also worsen preexisting pancreatic conditions such as pseudocysts in light of no other triggering factors for exacerbation? Our case proposes a novel insight: exacerbation of a regressing pseudocyst by a GLP-1 agonist in a patient without active pancreatitis risk factors, presenting with normal lab values. This case prompts an exploration of patient-specific factors, mechanistic underpinnings, alternative causes, long-term implications, and clinical considerations. Our study will navigate these nuances to provide a comprehensive understanding of this intricate relationship. Our goal is to bring forth this piece of literature in hopes that clinicians implement good screening practices for their patients who are on GLP-1 agonists and in hopes that the academic world of medicine can explore this phenomenon and advance our proposal further into a more established one.

## Case presentation

**Figure 1:**
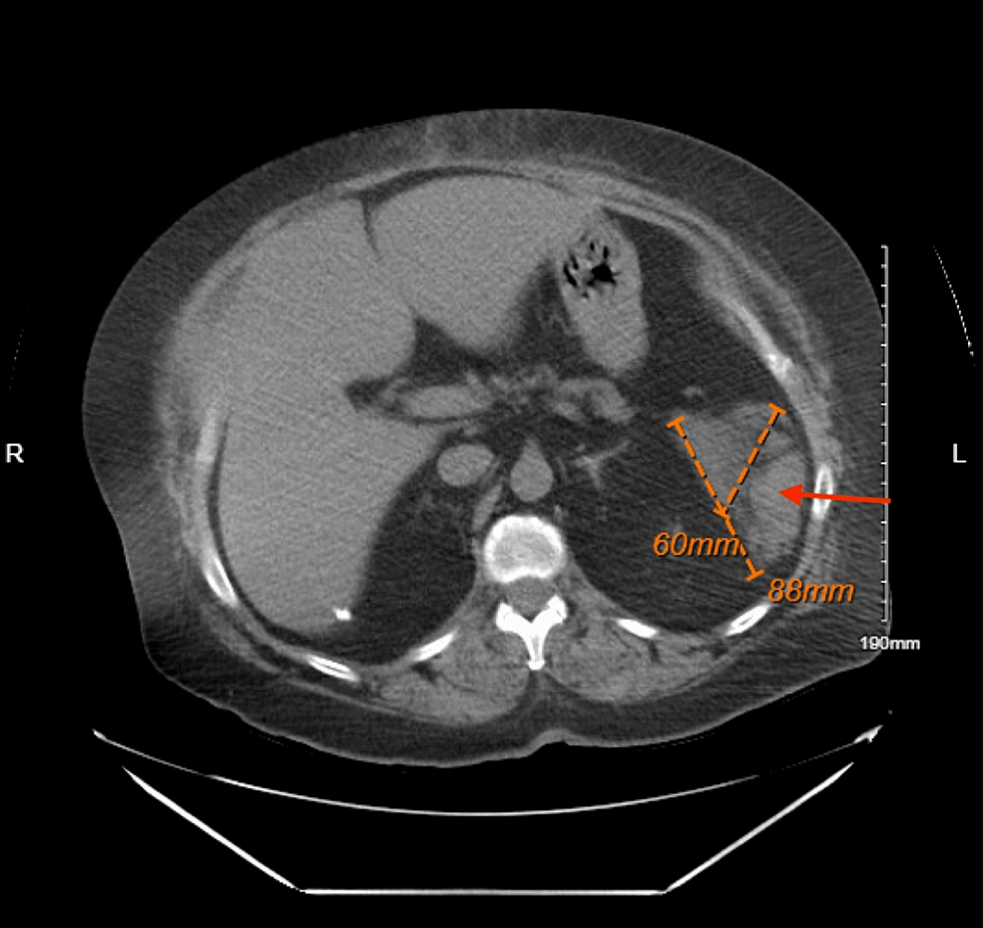
Computed tomography from a year prior to the patient's most recent visit depicting pseudocyst collection on the left side (red arrow) measuring 8.8 cm x 6.0 cm or 88 mm x 60 mm cm: centimeters; mm: millimeters

A 53-year-old female patient presented to the emergency room complaining of abdominal pain for the past five days. The pain was constant and involved the epigastric region with associated nausea, vomiting, and anorexia. The patient denied hematemesis, diarrhea, and melena. The pain radiated to her upper back. The patient noted that symptoms worsened with the intake of food. The quality of the pain was described as fullness in the abdomen and gnawing and burning sensation in the epigastric region. The patient stated that nothing she did to alleviate the pain and symptoms helped her. She thought symptoms were due to a medication she had just begun taking, a GLP-1 agonist. She was started on 0.25 mg, and it was increased to 1 mg six days before her symptoms started. Her past medical history was significant for type 2 diabetes mellitus with hyperglycemia, chronic obstructive pulmonary disease (COPD), ventral hernia repair, partial hysterectomy, and cholecystectomy. Post-hernia repair abdominal computed tomography (CT) scan from a year prior showed an existing pancreatic pseudocyst that measured 8.8 cm x 6.0 cm in conglomerate (Figure 1).

The patient regularly used a blood sugar meter, and her home medications are summarized in Table 1.

**Table 1:** Home medications mcg: microgram; mg: milligram

Medications
Tiotropium bromide 18 mcg
Cyanocobalamin 1,000 mcg
Hydroxyzine pamoate 100 mg
Ipratropium-albuterol 0.5 mg
Lamotrigine 200 mg
Levothyroxine 125 mcg
Metformin 1,000 mg
Omeprazole 40 mg
Tizanidine 4 mg
Trazodone 300 mg
Aspirin 81 mg
Bupropionhydrochloride 150 mg
Docusate sodium 100 mg
Insulin aspart U-100
Isosorbide mononitrate 15 mg
Letrozole 2.5 mg
Meclizine 12.5 mg
Metoclopramide hydrochloride 5 mg
Prochlorperazine maleate 5 mg
Topiramate 50 mg
Vilazodone 40 mg

The patient stated that she was compliant with her medications and takes them as prescribed. She is allergic to prasterone and sumatriptan. She denied any recent trauma or infection. She denies alcohol use but smokes tobacco half a pack to one pack a day.

On examination, the patient appeared uncomfortable; however, she was in no acute distress at the time of evaluation. She was overall well-nourished and well-hydrated. She was nontoxic in appearance. Vital signs included blood pressure of 110/69, heart rate of 80, respiratory rate of 18, and SpO2 of 91 % on room air as the patient has significant COPD. The abdominal exam was significant for slightly diminished bowel sounds. There was mild-to-moderate tenderness in the epigastrium without mass or rebound tenderness. Murphy's sign was negative. A large midline scar was appreciated from prior abdominal/hernia repair surgery. Our medical decision-making narrative and working diagnosis based on the patient's history and physical was that we suspected she was having abdominal pain due to her recent addition of GLP-1 agonist. Other differential diagnoses included acute pancreatitis, COPD exacerbation, uncontrolled type 2 diabetes mellitus with chronic kidney disease, poorly controlled gastroparesis, ileus, abdominal vessel vasculopathy, gastroesophageal reflux disease (GERD), and malignancy. We did appropriate tests to rule out the differential diagnoses like ordering labs and cancer antigen (CA) 19-9 to rule out malignancy and consulted Gastroenterology for further evaluation. Gastrointestinal endoscopy was also considered to rule out gastric bezoar. We presented our findings to the patient, and she agreed with our decision to admit her. Her laboratory investigations are recorded below (Table 2).

**Table 2:** Lab values WBC: white blood cells; Neut: neutrophils; Lymph: lymphocytes; Mono: monocytes; Eos: eosinophils; Baso: basophils; %: percentage; #: number; Auto: automated; BUN: blood urea nitrogen; AST: aspartate aminotransferase; ALT: alanine aminotransferase; uL: microliter; mmol/L: millimoles per liter; mg/dL: milligrams per deciliter; U/L: units per liter; H: high; L: low

Lab	Patient's values	Normal values
WBC	7.95 x 10^3^/uL	3.98-10.04 x 10^3^/uL
Neut % (Auto)	64.9 %	34.0-71.1 %
Lymph % (Auto)	23.0 %	19.3-51.7 %
Mono % (Auto)	7.3 %	4.7-12.5 %
Eos % (Auto)	4.0 %	0.7-5.8 %
Baso % (Auto)	0.5 %	0.1-1.2 %
Neut # (Auto)	5.16 x 10^3^/uL	1.56-6.13 x 10^3^/uL
Mono # (Auto)	0.58 x 10^3^/uL	0.24-0.86 x 10^3^/uL
Eos # (Auto)	0.32 x 10^3^/uL	0.04-0.36 x 10^3^/uL
Baso # (Auto)	0.04 x 10^3^/uL	0.01-0.08 x 10^3^/uL
Sodium	141 mmol/L	136-145 mmol/L
Potassium	3.4 mmol/L L	3.5-5.1 mmol/L
Chloride	109 mmol/L H	98-107 mmol/L
Anion gap	7.0 mmol/L	5.0-15.0 mmol/L
BUN	4 mg/dL L	7.0-18.0 mg/dL
Creatinine	0.93 mg/dL	0.55-1.02 mg/dL
BUN/creatinine ratio	4.3 ratio L	6.0-20.0 ratio
Glucose	136 mg/dL H	70-99 mg/dL
Calcium	9.0 mg/dL	8.5-10.1 mg/dL
AST	13 U/L L	15-37 U/L
ALT	23 U/L	14-59 U/L
Alkaline phosphatase	110 U/L	46-116 U/L
Amylase	30 U/L	25-115 U/L
Lipase	42 U/L L	73-393 U/L

**Figure 2:**
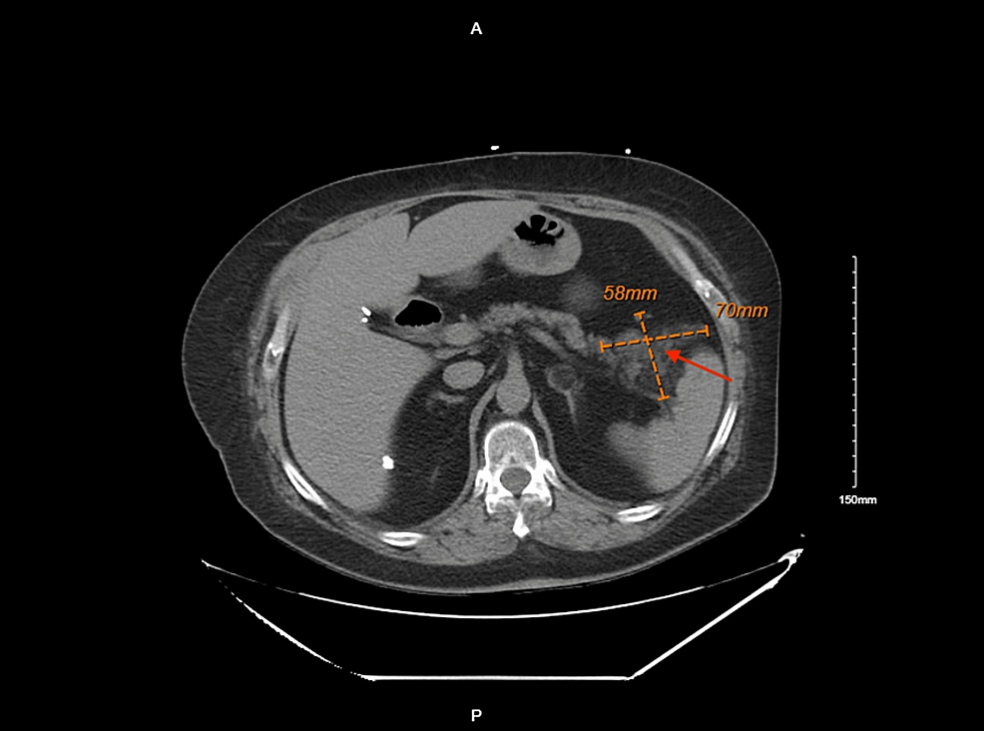
Computed tomography significant for a focal fluid collection (red arrow) seen at the tail of the pancreas measuring up to 7.0 cm x 5.8 cm or 70 mm x 58 mm in conglomerate cm: centimeters; mm: millimeters

We did further workup with a CT scan which was significant for a focal fluid collection seen at the tail of the pancreas measuring up to 7.0 cm x 5.8 cm in conglomerate which could have been related to pancreatic pseudocyst but was of unclear etiology (Figure 2).

**Figure 3:**
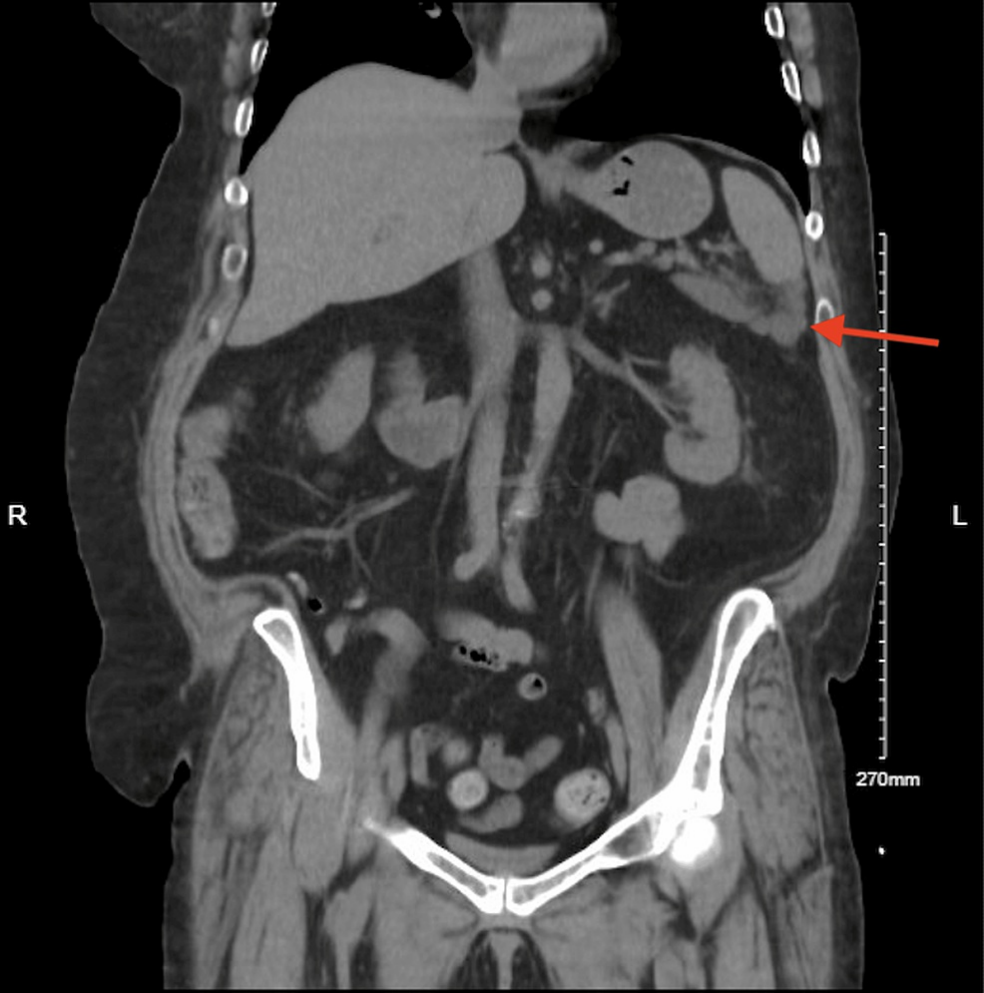
Absent gallbladder and biliary tree with pancreatic pseudocyst visualized distal to the spleen (red arrow)

The liver showed coarse calcifications noted in the right hepatic lobe. The gallbladder and biliary tree were surgically absent (Figure 3). 

**Figure 4:**
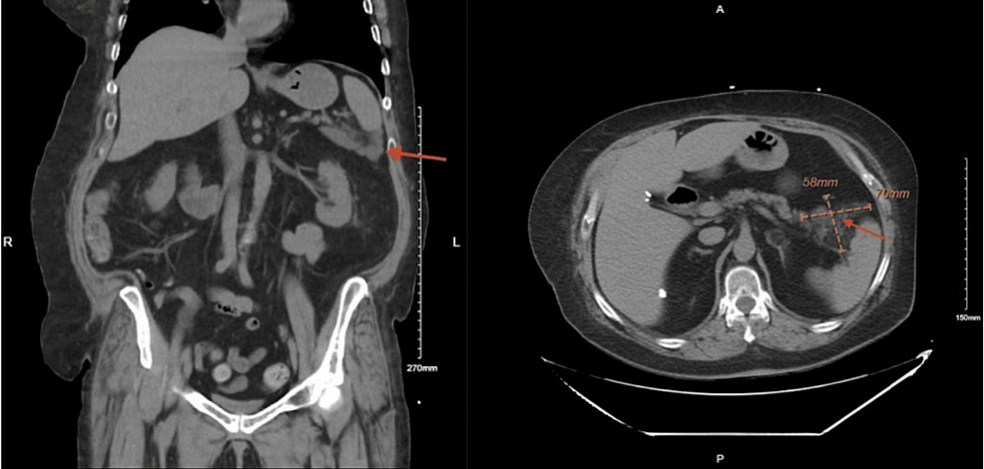
Left image: pancreatic pseudocyst visualized distal to the spleen (red arrow). Right image: surgical clips visualized on the right from prior cholecystectomy and pseudocyst on the left (red arrow)

All other structures were unremarkable (Figure 4).

**Figure 5:**
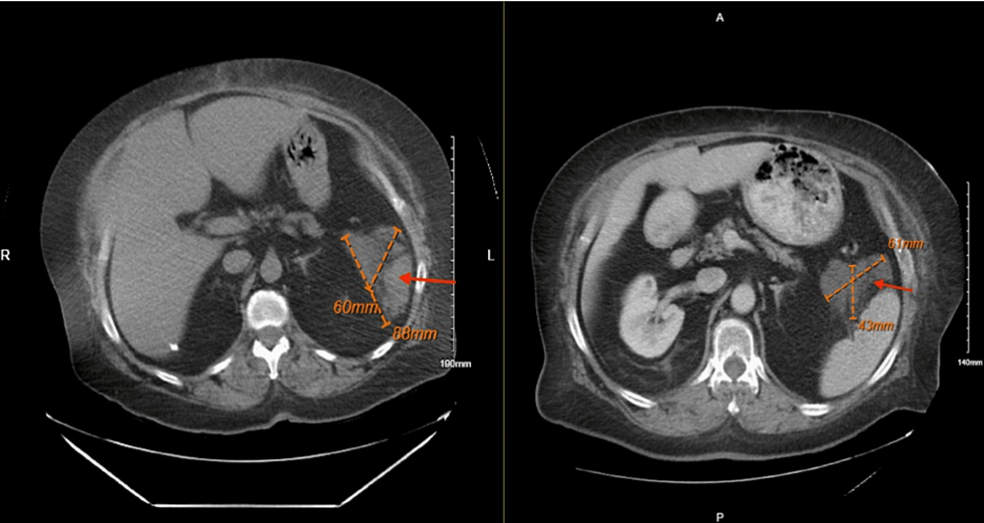
Left image: CT from a year prior with pseudocyst (red arrow) measuring 8.8 cm x 6.0 cm or 88 mm x 60 mm. Right image: most recent CT with pseudocyst (red arrow) measuring 6.1 cm x 4.3 cm or 61 mm x 43 mm cm: centimeters; mm: millimeters

We admitted the patient and continued to treat her conservatively with symptomatic management for a few days; however, our patient was still in pain, so we repeated the CT scan to monitor the pseudocyst. Comparing the CT scans from a year ago to her most recent one, it is apparent that her pseudocyst was decreasing in size, now measuring 6.1 cm x 4.3 cm in conglomerate, and there was no stranding around the pancreatic parenchyma ruling out pancreatitis as a cause for her abdominal pain (Figure 5). 

Her primary care physician followed up and discontinued GLP-1 agonist administration immediately. After reviewing the CT scans, we found that she had a regressing pseudocyst which was contributing to her symptomatology. She did not have any other risk factors at the time that could have possibly exacerbated her pseudocyst. Despite conservative management, her symptoms persisted, prompting a more invasive approach. General Surgery was consulted, and an esophagogastroduodenoscopy was performed in order to assess the pseudocyst. The patient was discharged a few days later upon resolution of her symptoms. 

## Discussion

After excluding all possible causes and any other alternative conditions that could have contributed to this patient's clinical presentation, the diagnosis of pancreatic pseudocyst with possible exacerbation secondary to GLP-1 agonist use in the absence of other aggravating factors was made. The results from one study explain the mechanistic insight of how elevations of amylase and lipase levels with the use of GLP-1 agonist were confirmed; however, the exact mechanism remains unclear [5]. One proposed mechanism from their study is that GLP-1 receptors are weakly expressed in acinar cells and GLP-1 agonists increase amylase secretion from these acinar cells. A result from the study has shown that four weeks of GLP-1 treatment increases protein synthesis in acinar cells and pancreatic mass, thus reflecting an increase in acinar cell mass without changes in ductal compartments or beta cell mass. Therefore, GLP-1 may enhance acinar cell protein synthesis including amylase and lipase. However, the effects of GLP-1 on exocrine function and pancreatic morphology have not been confirmed in non-human primates or humans [5]. Another hypothesis is that GLP-1 increases the permeability of the basolateral membrane of acinar cells, resulting in the enhanced transfer of pancreatic enzymes into the blood, improving glucose-induced insulin secretion by inducing GLP-1 receptors, but also releasing amylase and lipase enzymes into the blood [5]. This inappropriate release of pancreatic enzymes can induce drug-related pancreatic damage, resulting in parenchymal autodigestion [1,4]. However, this was not the case in our patient as her lab values at the time of admission showed normal amylase, lipase (a specific marker of pancreatic damage), aspartate aminotransferase (AST), calcium, and alkaline phosphatase (ALP) which are all atypical findings when investigating labs for pancreatitis (Table 2). Also, no stranding around the pancreas was appreciated on radiologic imaging for us to say she had a case of acute pancreatitis. This patient's case was purely an isolated symptomatic regressing pseudocyst that did not secrete any enzymes in the serum nor caused any localized tissue destruction. Prognosis is generally favorable in patients who have pancreatic pseudocysts as many regress spontaneously. In asymptomatic cases, pseudocysts are managed with supportive care, and symptomatic one's treatment typically involves endoscopic drainage, percutaneous drainage, surgical internal drainage, pseudocyst resection, and laparoscopic surgery [6]. In our case, the patient had already tried all the conservative therapies upon admission, none of which improved her symptoms. Her primary care physician had stopped the GLP-1 agonist, and she was then scheduled for an esophagogastroduodenoscopy to explore and assess the pseudocyst further. However, we did not drain her pseudocyst as it was regressing and not compressing local structures, an atypical finding for the patient's presentation. She was mobilized with supportive care and was discharged shortly thereafter upon improvement of her symptoms. There was no clear underlying cause for the patient's presentation, but stopping the GLP-1 agonist had shown improvement in her overall status. This led us to hypothesize that GLP-1 agonists could possibly aggravate pancreatic pseudocyst in rare cases if other risk factors are absent for exacerbation. This is why we present our case in hopes that this phenomenon can be further explored among a broader cohort, justified with the absolute pathophysiologic mechanism, and efficient clinical screening practices for similar cases can be implemented to prevent patients' adverse events.

## Conclusions

This case report emphasizes the importance of recognizing epigastric pain in a patient with pancreatic pseudocyst shortly after initiating GLP-1 agonist, despite normal enzyme levels, as a potential cause of exacerbation. Early diagnosis and appropriate management, along with close monitoring of subclinical/atypical symptoms in patients taking GLP-1 agonists, are vital to prevent further complications and to optimize patient outcomes. Healthcare professionals should maintain a high index of suspicion for pancreatic pseudocyst exacerbation in patients with a prior history of pseudocyst presenting with mid-epigastric pain and normal lab values, particularly when related to medications known to cause pancreatitis, like GLP-1 agonist, in the absence of other aggravating factors. Our goal is to present this case report in hopes that physicians implement proper screening practices for their patients who are on GLP-1 agonists and in hopes that with the help of academia, our proposed theory is further advanced into a more established one.

## References

[1] Wang GJ, Gao CF, Wei D, Wang C, Ding SQ (2009). Acute pancreatitis: etiology and common pathogenesis. World J Gastroenterol.

[2] Lankisch PG, Apte M, Banks PA (2015). Acute pancreatitis. Lancet.

[3] Habashi S, Draganov PV (2009). Pancreatic pseudocyst. World J Gastroenterol.

[4] Smits MM, Van Raalte DH (2021). Safety of semaglutide. Front Endocrinol (Lausanne).

[5] Saisho Y (2018). Incretin-based therapy and pancreatitis: accumulating evidence and unresolved questions. Ann Transl Med.

[6] Aghdassi AA, Mayerle J, Kraft M, Sielenkämper AW, Heidecke CD, Lerch MM (2006). Pancreatic pseudocysts--when and how to treat?. HPB (Oxford).

